# Facial hair enhancement with minoxidil—an off-label use

**DOI:** 10.1177/2050313X241231490

**Published:** 2024-02-23

**Authors:** Arveen Shokravi, Hanieh Zargham

**Affiliations:** University of British Columbia, Vancouver, BC, Canada

**Keywords:** hair, dermatology, facial hair, minoxidil

## Abstract

Minoxidil was first introduced in the 1970s as an anti-hypertensive medication. Hypertrichosis and scalp hair regrowth were noted by users, and the topical formulation of minoxidil was later approved by the Food and Drug Administration for androgenic alopecia and female pattern hair loss. Since then, minoxidil has been used off-label for various hair loss conditions and cosmetic outcomes. There are a multitude of informal reports on online communities presenting personal anecdotes regarding minoxidil’s effectiveness as a facial hair enhancement tool; however, this has been seldom discussed in the literature. In this report, we will present a case of identical twin males, one of which used topical 5% minoxidil for over a year on the beard and mustache area, while the other abstained from using the medication.

## Introduction

Minoxidil was first introduced in the 1970s as an anti-hypertensive medication. Hypertrichosis and scalp hair regrowth were noted by users, and the topical formulation of minoxidil was later FDA-approved for androgenic alopecia and female pattern hair loss. Since then, minoxidil has been used off-label for various hair loss conditions such as alopecia areata, chemotherapy-induced alopecia, frontal fibrosing alopecia, monilethrix, and telogen effluvium, and for cosmetic outcomes such as eyebrow and facial hair enhancement.^1^

There are a multitude of informal reports on online communities presenting personal anecdotes regarding minoxidil’s effectiveness as a facial hair enhancement tool. However, this has been seldom discussed in the literature, with one randomized trial showing minoxidil’s efficacy for beard enhancement, and a handful of case reports discussing the matter.

In this report, we will present a case of identical twin males, one of which used topical 5% minoxidil for over a year on the beard and mustache area, while the other abstained from using the medication. The goal of this study is to educate practitioners on this common practice, and thus, we will review minoxidil’s various formulations, side effect profile, utility, and other potential modalities that can be used concurrently to enhance its effectiveness.

## Report

As established by observation, the twins were identical/monozygotic twin males with no existing medical conditions. One twin applied approximately 1.5 g (¾ of a cap) of 5% topical minoxidil foam once daily to the beard and mustache area, while the other refrained from using the medication. Subjects reported having similar, if not nearly identical, facial hair density and distribution prior to the initiation of the medication.

**Figure 1.**
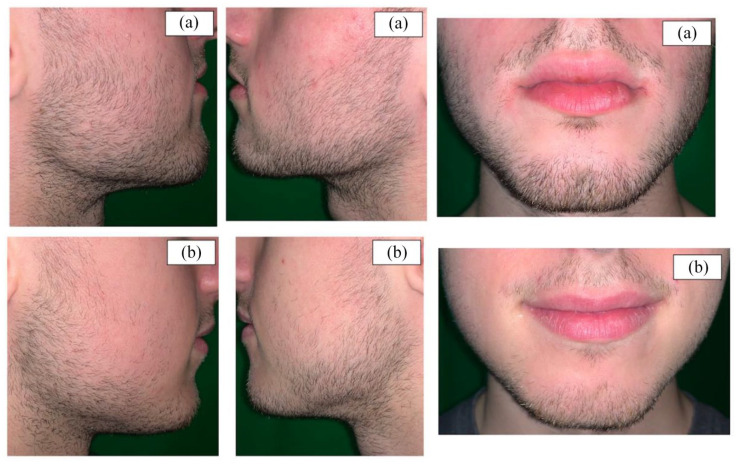
(a) Minoxidil treated subject after 16 months of once daily 1.5 g 5% minoxidil foam application. (b) non-minoxidil treated subject. All photos taken at the same time after 10 days of hair growth post-shave.

At the 1 month mark the minoxidil-treated patient noted the presence of new, finer, lighter colored hairs, and by the 2-month mark noted a modest increase in facial hair density. The patient reported shedding of the hairs in the beard area approximately 3 months after initiating minoxidil, but noted that eventually the facial hair growth recovered and continued to progress. This shedding process has been described on the scalp; it is speculated that it represents the hairs in the telogen phase shedding early before beginning the anagen phase.^2^ Following the conclusion of the shedding phase, facial hair growth continued to advance gradually. As seen in Figure 1, a greater hair count and density in both the beard and mustache areas can be appreciated in the minoxidil-treated twin after 16 months of use.

It is worth highlighting that the patient initially used liquid minoxidil solution for the first 3 weeks, but switched to the foam formulation as the former caused significant dry flaky skin. The patient reported no major side effects and minimal mild side effects with foam minoxidil use. Mild skin dryness, hypertrichosis on the ears and forehead, and an increase in body hair (chest, abdomen, forearms, and legs) were noted.

## Discussion

Topical minoxidil comes in two forms: liquid solution and foam, although foam is more convenient as it dries more rapidly. Topical minoxidil can also be found in different concentrations (most commonly 2% and 5%, although higher concentrations have been used). The oral formulation of the medication also exists but has a greater side effect risk profile.^1^ On the scalp, the uptake of the drug is approximately 50% 1 h after application and 75% after 4 h. Due to its half-life of 22 h, minoxidil should be applied twice daily for optimal results.^3^

Minoxidil’s effect on hair growth is primarily due to its metabolite, minoxidil sulfate. The sulfotransferase enzyme is responsible for the catalysis of this reaction. The exact mechanism of action of minoxidil is unknown, although it is postulated that minoxidil’s vasodilatory properties contribute to the positive hair growth effects. Minoxidil has also been shown to affect cell growth, prostaglandin synthesis, collagen synthesis, and VEGF expression.^2^

Minoxidil’s ability to grow scalp hair has been extensively discussed in the literature, although there has only been one randomized control study presenting its potential for facial hair enhancement. The randomized, double-masked, placebo-controlled study showcased minoxidil’s ability to enhance facial hair count in a cohort of 48 men aged 20–60. Application of 0.5 mL of 3% minoxidil liquid solution twice daily led to a statistically significant increase in hair count within the 16-week trial.^4^

It is important to note that discontinuation of minoxidil on the scalp causes regression of the hairs gained and an eventual return to the pre-treatment state, although it is unknown whether this phenomenon applies to facial hair.^2^ Some informal reports suggest that the terminal facial hairs gained while on minoxidil persist with the cessation of the drug while the vellus hair do not, but to our knowledge, there have been no studies conducted on the matter. The potential for the permanence of minoxidil-induced facial hair is intriguing and may be due to a concept known as the “androgen paradox,” where androgens will stimulate or suppress hair growth depending on the body site the follicle is located (as seen in the beard and scalp in males respectively).^5^ The reason for this can be attributed to the intrinsic variation among hair follicles in different body areas, potentially due to distinctions in the post-androgen receptor cascade and/or differences in expression of androgen receptor and 5α-reductase type 2 genes, which are presumed to be established during embryogenesis.^6^

The most frequently reported side effects of topical minoxidil are allergic or contact dermatitis,^2^ along with pruritis, headache, and hypertrichosis. Palpitations and tachycardia have also been reported as potential side effects of topical minoxidil;^1^ therefore, patients with cardiovascular disease should be more closely monitored when using the medication.

It is important to note that anecdotal reports suggest that response to minoxidil treatment can vary among patients. While some individuals experienced substantial improvements in facial hair growth within weeks to months, others have reported making minimal progress after 2+ years of use. Consequently, other modalities can be used alongside minoxidil to enhance its effectiveness in the “non-responder” population or for those who want to accelerate the progression timeline. Topical tretinoin can be incorporated alongside minoxidil for those with low responsiveness to the drug. Tretinoin has been shown to enhance minoxidil’s activity. Topical tretinoin upregulates the expression of the follicular sulfotransferase enzymes, allowing for more formation of the active product, minoxidil sulfate.^7^ Minimally invasive procedures such as microneedling, dermarolling, or dermastamping can also be used alongside minoxidil. Microneedling combined with concurrent 5% minoxidil use has been shown to increase hair count in hair loss conditions more than either minoxidil or microneedling monotherapy.^8^ However, no literature has been published showing the optimal needle depth and needling frequency for facial hair growth.

## Conclusion

The notable hair growth stimulation properties of minoxidil, coupled with its relatively favorable side-effect profile, establish it as a valuable intervention for those seeking to enhance facial hair growth. Clinicians should be aware of this off-label use and consider potential side effects, different dosage and formulation options, and modalities that can be used alongside minoxidil when discussing treatment options with patients. The numerous anecdotal reports and limited literature available on this topic emphasize the need for more comprehensive studies to guide clinical decision-making and address current knowledge gaps.
